# Musashi-1 regulates AKT-derived IL-6 autocrinal/paracrinal malignancy and chemoresistance in glioblastoma

**DOI:** 10.18632/oncotarget.9890

**Published:** 2016-06-07

**Authors:** Hsiao-Yun Chen, Liang-Ting Lin, Mong-Lien Wang, Shu-Hsien Lee, Ming-Long Tsai, Chi-Chang Tsai, Wei-Hsiu Liu, Tzu-Chien Chen, Yi-Ping Yang, Yi-Yen Lee, Yuh-Lih Chang, Pin-I Huang, Yi-Wei Chen, Wen-Liang Lo, Shih-Hwa Chiou, Ming-Teh Chen

**Affiliations:** ^1^ Institute of Clinical Medicine, National Yang-Ming University, Taipei Veterans General Hospital, Taipei, Taiwan; ^2^ Institute of Pharmacology, National Yang-Ming University, Taipei Veterans General Hospital, Taipei, Taiwan; ^3^ School of Medicine, National Yang-Ming University, Taipei Veterans General Hospital, Taipei, Taiwan; ^4^ Graduate Institute of Medical Sciences, National Defense Medical Center, Department of Neurological Surgery, Taipei Veterans General Hospital, Taipei, Taiwan; ^5^ Department of Medical Research, Taipei Veterans General Hospital, Taipei, Taiwan; ^6^ Cancer Center, Taipei Veterans General Hospital, Taipei, Taiwan; ^7^ Division of Oral and Maxillofacial Surgery, Department of Stomatology, Taipei Veterans General Hospital, Taipei, Taiwan; ^8^ Department of Neurosurgery, Neurological Institute, Taipei Veterans General Hospital, Taipei, Taiwan

**Keywords:** Musashi-1, apoptosis, IL-6, chemoresistance, GBM

## Abstract

Glioblastoma multiform (GBM) is one of the most lethal human malignant brain tumors with high risks of recurrence and poor treatment outcomes. The RNA-binding protein Musashi-1 (MSI1) is a marker of neural stem/progenitor cells. Recent study showed that high expression level of MSI1 positively correlates with advanced grade of GBM, where MSI1 increases the growth of GBM. Herein, we explore the roles of MSI1 as well as the underlying mechanisms in the regulation of drug resistance and tumorigenesis of GBM cells. Our results demonstrated that overexpression of MSI1 effectively protected GBM cells from drug-induced apoptosis through down-regulating pro-apoptotic genes; whereas inhibition of AKT withdrew the MSI1-induced anti-apoptosis and cell survival. We further showed that MSI1 robustly promoted the secretion of the pro-inflammatory cytokine IL-6, which was governed by AKT activity. Autonomously, the secreted IL-6 enhanced AKT activity in an autocrine/paracrine manner, forming a positive feedback regulatory loop with the MSI1-AKT pathway. Our results conclusively demonstrated a novel drug resistance mechanism in GBM cells that MSI1 inhibits drug-induced apoptosis through AKT/IL6 regulatory circuit. MSI1 regulates both cellular signaling and tumor-microenvironmental cytokine secretion to create an intra- and intercellular niche for GBM to survive from chemo-drug attack.

## INTRODUCTION

Brain tumors, according to World Health Organization (WHO), can be categorized into four grades from I to IV [1]. Glioblastoma multiforme (GBM), graded IV, is one of the most frequent and aggressive human primary brain tumors [2], which remain highly undifferentiated [3]. Surgical dissection combining chemotherapy or radiotherapy is presently the general guideline of GBM in curative intent; however, therapeutic resistance and recurrence are still a unsolved conundrum [4]. Consequently, GBM cases have a median survival of approximately 15 months. Therefore, understanding the key mechanism involved in drug and therapeutic resistance is of critical in searching novel effective GBM treating strategy.

Musashi-1(MSI1) is one of neural stem cell marker that is highly expressed in the central nervous system. The primary structure and the expression pattern of MSI1 have been recognized among species in nematodes, Drosophila as well as ascidian [2]. In mammalian, MSI1 is believed to be a marker of neural stem cells and progenitor cells. Okano et al. firstly proved that MSI1 has multiple functions in regulating cell fate decision and maintaining the stem cell state [5]. Additionally, highly expressed MSI1 are associated with high-grade glioma; as a result, the prognostic potential of MSI1 has been approved by survival analysis [6]. Moreover, knockdown of MSI1 was found to promote tumor regression and radiation-induced colon cancer cells apoptosis, indicating that MSI1 plays important roles in cancer cell proliferation, inhibition of apoptosis and modulating of tumor progression [7]. The underlying mechanism had been implied by the MSI1-mediated inhibition of Numb and the downstream activation of PI3K/AKT pathway in glioma cells [8]. Although several functional aspects of MSI1 have been characterized, there are still many issues in the molecular mechanism, especially the role in tumor resistance and tumor pathogenicity, remain questioned.

AKT, also known as protein kinase B, is a serine/threonine protein kinase that plays a critical role in cell growths, cell survival, apoptosis, and tumor progression by activating a series of different downstream signaling pathways [9]. In some tumor, constitutively activated AKT is associated with increased drug resistance and cell survival [10]. A variety of AKT inhibitor have been shown to prevent tumor cell growth and induce apoptosis both *in vitro* and *in vivo* [11]. The pathway leading to AKT activation involves receptor tyrosine kinase including PI3K (phosphatidylinositol 3-kinase) [12]. Many pattern recognition receptors, growth factor receptors and cytokine receptors are able to activate PI3K, and thereby activate AKT [13]. Recently studies have shown that the AKT signaling is involved in regulating the inflammatory response and modulating of cancer cell development and anti-apoptosis [14].

Inflammatory cytokines have been found as critical mediator in GBM microenvironment, which predominantly regulate tumor growth, metastasis, and drug resistance [15]. Among the well-characterized cytokines, interleukin-6 (IL-6) is one of the important inflammatory factors which regulates cell proliferation and anti-apoptosis [16]. Previous studies that IL-6 are reported to overexpress in breast, liver, colon and brain tumor. Moreover, IL-6 activates several pro-proliferation and survival proteins in order to stimulate tumor cell growth [17]; whereas, the inhibition of IL-6 signaling was shown to reduce both glioma size and aggressiveness [18]. For instance, IL-6-induced PI3K/AKT activation was essential for anti-apoptotic signaling cascade, which has long be linked to therapeutic resistance [19]. Thus, the aim of this study was to draw the detail mechanism of MSI1 in regulating chemo-resistance and to determine whether MSI1 affects apoptotic events through IL-6 regulatory circuit. Indeed, our results indicated that MSI1 activates AKT with phosphorylation and further induces IL-6 biogenesis and secretion while drug is encountered. Inhibition of AKT activation in MSI1-overexpressed cells greatly reduced the autocrinal/paracrinal IL-6 and increased in the number of apoptotic cells upon chemo-drug stimulation. In this study, we revealed MSI1 plays an important role in AKT activation and IL-6 secretion in response to chemo-drug in GBM cells, which eventually contributes to a dynamic interaction between proinflammatory circuits, chemoresistance, and tumor recurrence.

## RESULTS

### Musashi-1 regulated *in vitro* tumorigenic ability of GBM to resist chemodrug-induced cell death

**Figure 1:**
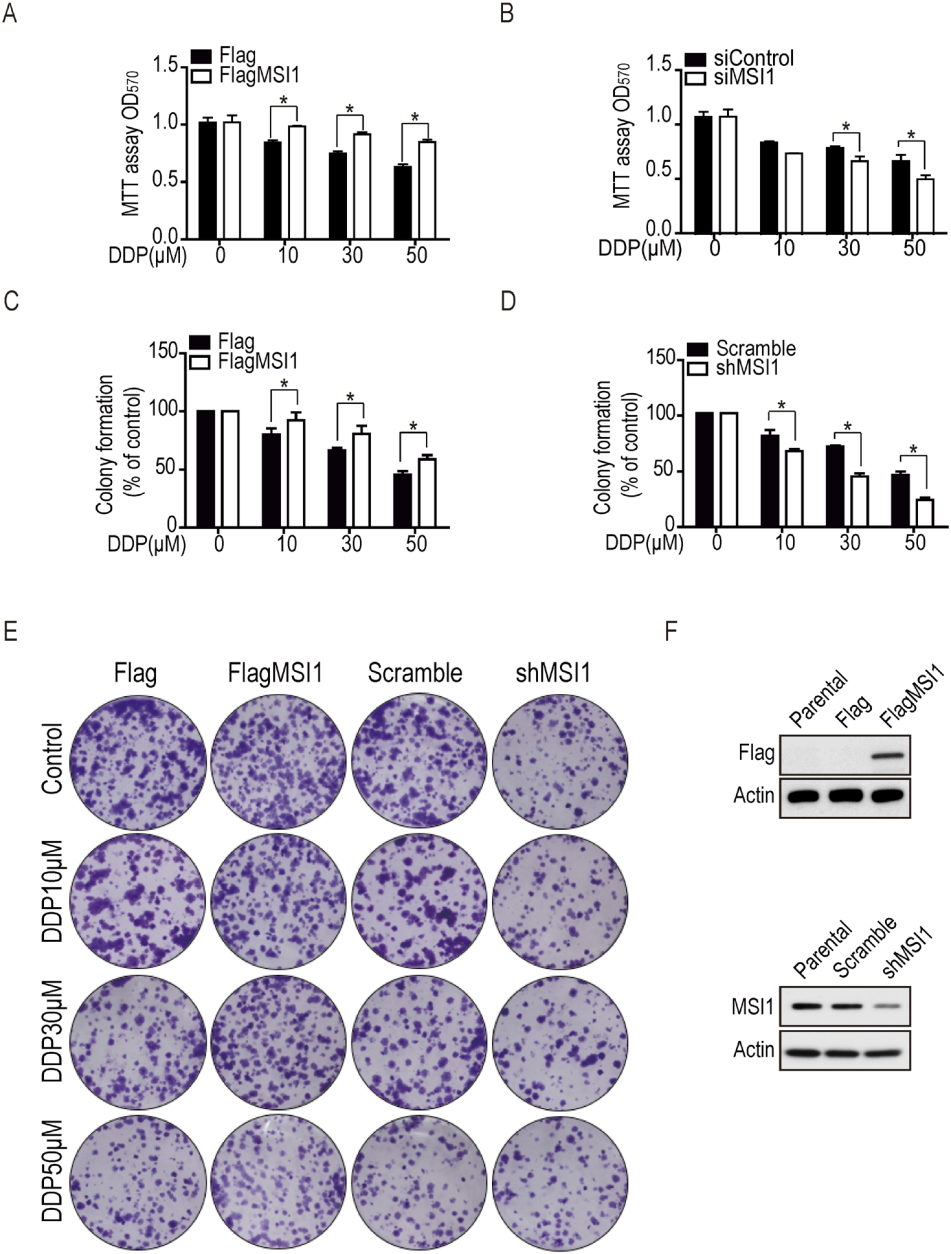
Musashi-1 enhanced cell viability and colony formation under DDP treatment in GBM cells **A.** The Flag-tagged Musashi-1 (FlagMSI1) and empty vector (Flag) transfected stable cells were established in 05MG GBM cell line and subjected to a dose-course DDP treatment. The cell viability was determined by MTT assay. **B.** Control (siControl) and siMusashi-1 (siMSI1) siRNA transfected 05MG cells were subjected to MTT viability assay under dose-course DDP treatment. **C.** 05MG-FlagMSI1 and 05MG-Flag cells were treated with different concentration (from 0 to 50 μM) of DDP for 24 hours, followed by a colony formation assay incubated for 10 days allowing the survived cells forming colonies. Data represent the mean ± S.D. of three independent experiments performed in triplicate. * *P*<0.05 vs control cells. **D.** Musashi-1 knockdown (shMSI1) cells and scrambled shRNA (scramble) transfected control cells were established in 05MG GBM cell line and subjected to a colony formation assay as described in C. **E.** The formation of colonies in C and D was photographed. **F.** Protein expression level for MSI1 in MSI1-overexpressed cells and knockdown cell lines by Western blotting.

Accumulated reports have indicated that MSI1 is able to promote drug resistance and cell survival through various signaling pathways in glioma [8, 14–16], but the downstream regulators still remain debating. To address the role of MSI1 on drug resistance in GBM cells, we initially evaluated the cell viability in 05MG GBM cell line with either over-expressed or knockdown MSI1 expression in the presence or absence of chemotherapeutic agents. Cells was treated with cisplatin (DDP) in various concentration for 24 hrs; MTT assay was performed to observed cell viability. The OD_570_ values showed no significant difference on cell survival rate between Flag-control and MSI1-overexpressed cells; while 50 μM DDP led to around 35% cell death in Flag-control cells but only 15% cell death in MSI1-overexpressed cells (Figure 1A). Consistently, this effect was conversely displayed in MSI1-knockdown cells, where 50 μM DDP led to 50% cell death in MSI1-knockdown cell but only 30% in parental cells (Figure 1B). The same result was also observed with ATO treatment (Suppl. Figure 1A and Suppl. Figure 1B), suggesting that MSI1 prevents GBM cells from chemotherapy-induced cells death. Next to evaluate whether MSI1 promotes cells survival during DDP treatment in GBM cells, the colony formation assay with a dose-course treatment of DDP was performed (Figure 1C-1E). As expected, treatment of 50 μM DDP on Flag-control cells yielded a surviving fraction of 50%; while the same treatment on MSI1-overexpressed cells led to a surviving fraction of 70% (Figure 1C). On the other hand, parental cells showed 50% surviving fraction under 50 μM DDP treatment, while MSI1-knocked cells showed only 30% (Figure 1D). The same result was also observed with ATO treatment (Suppl. Figure 1C-1E). These data suggested that MSI1 promotes cells survival under chemodrug-mediated cell dysfunction. According to our finding, MSI1 enhanced cell survival and preserved the tumorigenesis capability upon chemodrugs treatment in GBM cells. Musashi-1 enhanced chemoresistance of GBM cells through repressing apoptotic pathway.

### Musashi-1 enhanced chemoresistance of GBM cells through repressing apoptotic pathway

**Figure 2:**
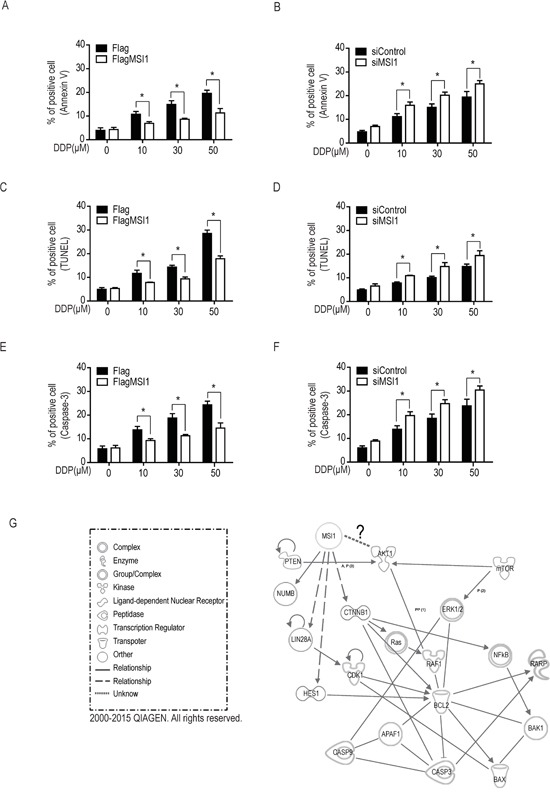
Musashi-1 mitigated DDP-induced apoptosis in GBM cells **A-F.** MSI-overexpressed (A, C, E) and knockdown (B, D, F) cells were treated by a dose-course of DDP for 24hrs. The apoptotic cells were identified by the staining of AnnxinV (A-B), terminal deoxynucleotidyl transferase dUTP nick end labeling (TUNEL; C-D, and activated caspase-3 (E-F) and quantified by flow cytometer. Data represent the mean ± S.D. of three independent experiments performed in triplicate. * P<0.05 vs control cells. **G.** The identified significant cell survival pathway from the mRNA array analysis are organized in a connectivity network constructed by Ingenuity IPA software. Both MSI1 and AKT were the core factors in this network.

It has been reported that MSI1 could regulate drug-induced apoptosis via several signaling cascade [17–19]. To further explore the underlying mechanism of MSI1-dependent anti-apoptotic pathway, we performed assays, including AnnexinV, TUNEL and active caspase-3 for evaluating the apoptotic events in drug treated GBM cells. As shown in Figure 2A, the AnnexinV-positive signals in Flag-control cells was similar to MSI1-overexpressed cells in normal condition. 50 μM DDP leaded about 18.5% AnnexinV-positive cells in Flag-control cells death, however only caused 11.5% cell death in MSI1-ovexpressed cells. Moreover, TUNEL was used to investigate DDP-facilitate DNA damage, as show in Figure 2C, 50 μM DDP leaded about 28.5% TUNEL-positive cells in Flag-control cells death, but only caused 17.5% TUNEL-positive cells in MSI1-ovexpressed cells. Science cleaved caspase-3 is known to play vital roles to modulated cell apoptosis pathway, therefore we also investigated the caspase-3 positive cells. In Figure 2E, 50 μM DDP leaded about 25% cleaved caspase-3 positive cells in Flag-control cells death, however only caused 10.5% cleaved caspase-3 positive cells in MSI1-ovexpressed cells. Remarkably, the anti-apoptotic effect was withdrawn by the suppression of MSI1 expression (Figure 2B, 2D, 2F). Not only DDP but ATO-induced apoptosis could be eliminated by the overexpression of MSI1 (Suppl. Figure 2), suggesting that MSI1-preferentially leads to an anti-apoptotic fashion in response to chemotherapeutic drugs in GBM cells. Based on our data in Figure 1 and Figure 2, MSI1 inhibited DDP-induced cell apoptosis, including reduced pro-apoptosis protein expression, attenuates AnnexinV positive cells, and decreased TUNEL positive cells. Moreover, the MSI1-dependent enhancement of cell survival did not involve enhanced proliferation rate (Figure 2). Most importantly, all of the MSI1-dependent anti-apoptotic effects were only shown under DDP treatment. To further elucidate the potential pathways and factors involved in MSI1 mitigated chemodrug-induced apoptosis, we used Ingenuity Pathway Analysis (IPA, Qiagen Inc.) as a bioinformatic platform to identify the possible network shared between MSI1 and other apoptosis-related candidates. As shown in Figure 2G, MSI1 and AKT were found to jointly decrease apoptosis as well as survival regulatory network. Regarding that AKT was reported to activate BAD and prevents caspase-3 from cleaving [20], we thereby illustrated the potential regulatory trend between MSI1 and AKT activation in response to chemotherapy.

### Musashi-1 regulated AKT activation to mitigate drug-induced apoptosis

**Figure 3:**
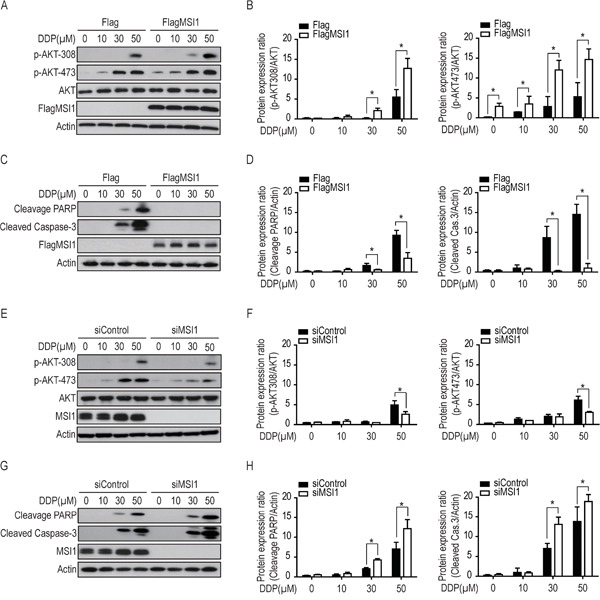
Musashi-1 enhanced AKT phosphorylation and reduced the levels of cleaved-caspase-3 and cleavage-PARP under DDP treatment **A-D.** 05MG-FlagMSI1 and 05MG-Flag cells were treated with different concentration (from 0 to 50 μM) of DDP for 24 hours. Total lysates were analyzed by Western blot to assess the level of phosphorylated AKT (A-B), and cleaved PARP / Caspase-3 (C-D). The levels of phosphorylated AKT (B) and cleaved PARP / Caspase-3 (D) were quantified and standardized with the levels of total AKT and actin, respectively. **E-H**. 05MG-siMSI1 and 05MG-siControl cells were treated with different concentration of DDP for 24 hours, followed by Western blot analysis and quantification as described in A-D. Data represent the mean ± S.D. of three independent experiments performed in triplicate. * P<0.05 vs AKT or Actin.

A recent report has indicated that MSI1 is capable of enhancing the growth and/or survival of glioma cells through Notch and PI3K/AKT signaling pathway [8]. However, whether MSI1 involves in AKT-dependent apoptotic pathway is still an open question. Hence, we turned our attention to test whether MSI1 predominantly activates AKT during drug treatment. Immunoblot revealed that AKT was enhanced by two phosphorylation sites (Thr308 and Ser473) of which MSI1-overexpressed GBM cells were significantly sensitive than that of Flag-control cells in a dose-dependent manner (Figure 3A and 3B). Overexpression of MSI1 suppressed the DDP-induced activation of PARP and Caspase-3 (Figure 3C and 3D). Similarly, in the case of ATO treatment, ectopic expression of MSI1 also increased AKT phosphorylation at the two sites and decreased the activation of both PARP and Caspase-3 (Suppl. Figure 3), indicating that MSI1 overexpression was capable of engagement against cytotoxic stress. Consistently, knockdown of MSI1 by siRNA resulted in suppressed AKT phosphorylation and increased PARP and Caspase-3 activity in GBM cells, in comparison to the control (Figure 3E-3H). As a result, significant lower level of cleaved caspase-3 and PARP in response to chemotherapy in MSI1-overexpressed GBM cells have drawn a potential regulatory relationship between MSI1 and AKT as well as the apoptosis signaling. Inhibition of AKT-phosphorylation blocks MSI1-induced chemoresistance in GBM cells.

**Figure 4:**
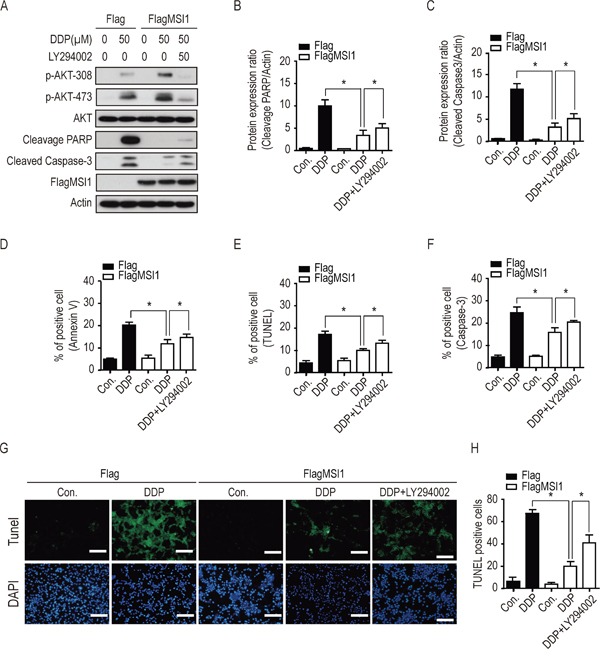
Inhibition of AKT activity blocked the suppressive effect of Musashi-1 on DDP-induced apoptosis **A-F.** 05MG-FlagMSI1 and 05MG-Flag cells were pretreated with 50 μM of LY294002 or vehicle for 3 hours, followed by 50 μ0e addtreatment for additional 24 hours. **A.** Total cellular extracts were analyzed by Western blot analysis. **B.** Quantified cleaved PARP protein levels in A were standardized with actin level. **C.** Quantified cleaved Caspase-3 protein levels in A were standardized with actin level. Data represent the mean ± S.D. of three independent experiments performed in triplicate. * P<0.05 vs actin. **D-F.** Percentage of apoptotic cells were quantified by Flow cytometry in Annexin V (D), TUNEL (E), and Caspase-3 (F) assay. Data represent the mean ± S.D. of three independent experiments performed in triplicate. **G.** Green fluorescence indicates TUNEL positive cells. Top panel: apoptotic cells stain with FITC; bottom panel: nuclei stained with DAPI. **H.** Quantitative evaluation of TUNEL positive cells. Eight images were analyzed in five 05MG sections from each group. Data represent the mean ± S.D. of two independent experiments performed in duplicate. * P<0.05 vs Flag control or FlagMSI1 add DPP. Scale Bar =100 μm.

Since previous reports have indicated the crucial role of active AKT in promoting drug resistance and apoptosis pathway [12, 20, 21], we further studied if AKT activation is essential in MSI1-induced chemoresistance. We inhibited AKT phosphorylation by LY294002 (PI3K inhibitor) and analyzed the protein levels of active caspase-3 and cleaved-PARP by Western blot. Compared with Flag-control GBM cells, DDP-induced expression of active caspase-3 and cleaved-PARP were significantly decreased in MSI1-overexpressed GBM cells. Interestingly, the addition of LY294002 partly restored the expression of active caspase-3 and cleaved-PARP under DDP treatment in MSI1-overexpressed GBM cells (Figure 4A-4C). Similar results were also obtained by ATO treatment (Suppl. Figure 5A-5C), suggesting activated AKT is essential for MSI1-dependent suppression of PARP and Caspase-3 activity.

Next, we assessed the percentage of apoptotic cells by the ratio of AnnexinV/PI, TUNEL/PI, and active caspase-3/PI with flow cytometry. As show in Figure 4D to 4H and Suppl. Figure 4, both DDP and ATO induced apoptosis in parental GBM cells; compared with the Flag-control cells (20% AnnexinV-positive cells, 18.5% TUNEL-positive cells and 22% cleaved casapase-3 positive cells), chemodrug-induced apoptosis was significantly decreased in MSI1-overexpressed GBM cells (11% AnnexinV-positive cells, 10% TUNEL-positive cells and 14% cleaved casapase-3 positive cells). Interestingly, under DDP or ATO treatment, inhibition of AKT by LY294002 elevated the percentage of apoptosis in MSI1-overexpressed cells (17% AnnexinV-positive cells, 12.5% TUNEL-positive cells and 20% cleaved casapase-3 positive cells), compared with that in MSI1-overexpressed cells without LY294002 treatment (Figure 4D-4H; Suppl. Figure 4D-4F). These data implied that activated AKT mediates MSI1-dependent induction of chemoresistance in GBM cells, and that inhibition of AKT activity sensitizes GBM cells to chemotherapeutic treatment.

### Autocrine/paracrine biogenesis was enriched in DDP-treated GBM cells via a Musashi-1/AKT signaling cascade

**Figure 5:**
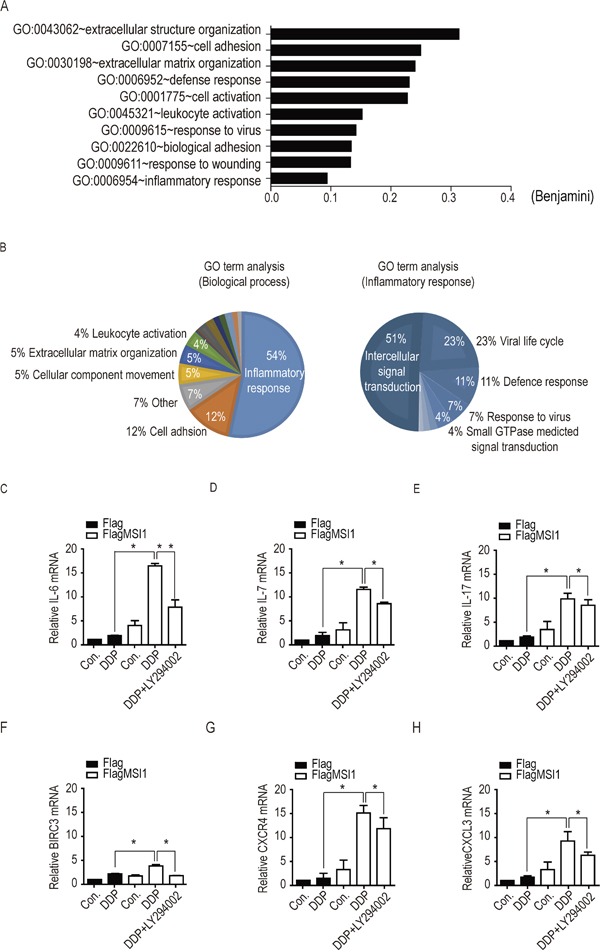
Identification of mRNA expression profiles in Musashi-1 overexpressed cells under DDP treatment **A.** Gene ontology (GO) enrichment analysis was conducted by DAVID software according to the category of biological processes. Benjamini ≤ 0.05 is selected as interesting GO. The GO accession, name, and the corresponding p-value were shown in the graph. **B.** Top 50 GO classes were analyzed by the visualization software REVIGO (REduce and VIsualize GO; (Supek et al., 2011); http://revigo.irb.hr/). The number on each pie shows the corresponding GO term and its percentage. GO ID number and log (p-value) of each GO term is indicated. **C-H.** Cells were pretreated with 50 μM of LY294002 or vehicle for 3 hours, followed by 50μM DDP treatment for 24 another hours. Total cellular extracts were analyzed by real-time PCR to validate the mRNA levels of IL-6, IL-7, IL-17, BICR3, CXCR4 and CXCL3. The relative expression levels of each mRNA was standardized with actin mRNA level.

Elevated evidences have demonstrated the dominant role of pro-inflammatory circuits and tumor-microenvironment interaction within tumorigenicity, drug resistance, and tumor recurrences [22–25]. Several secreted factors, including tumor-associated inflammatory cytokines were shown to regulate autocrine/paracrine signaling in mediating the malignant transformation of tumor cells including chemoresistance [26–28]. To explore the potential targets involving in MSI1-driven tumor-resistance in malignant glioma, we sought to *in silico* analyze the global gene expression profile to explore the impact of MSI1 overexpression in GBM cells. Comparing MSI1-overexpressing cells with Flag-control cells, we screened out the expression level lower than 1.5 log2 ratio (subtotal 989 genes, of which 564 genes were up-regulated and 425 genes were down-regulated) and subjected to DAVID bioinformatics resource (https://david.ncifcrf.gov/summary.jsp) and REVIGO (http://revigo.irb.hr/revigo.jsp) for Gene Ontology (GO) enrichment analysis. Furthermore, using Benjamini-Hochberg procedure, the immune inflammatory response and pro-inflammatory cytokines-related pathways, including CCL2, CCL3, CCL4, CXCR4, CXCR12, IL2, IL-6, IL-7, IL17, IL23, and cIAP2, were found highly expressed in our results. The annotated gene sets were identified with the lowest false discovery rate (FDR) (Figure 5A). Moreover, the GO: biological processes accession, name, and the corresponding p-value (Fisher extract test) were clustered together in superclusters of related terms (Figure 5B), suggesting the importance of pro-inflammatory events in MSI1-modulated GBM cells. Among the genes highlighted, we found the DDP-induced mRNA level of IL-6, IL-7, IL-17, cIAP2, CXCR4 and CXCL12 were highly enriched while MSI1 up-regulated (Figure 5C-5H). Furthermore, the inactivation of AKT partly withdrew the enrichment, of which IL-6 showed the most significant change in response to LY294002 (Figure 5C-5H). Therefore, these results indicated that the biogenesis of IL-6 could lead to activation of AKT under chemotherapeutic stress.

### Musashi-1/AKT-mediated secretion of pro-inflammatory IL-6 autonomously compromises cisplatin-induced apoptosis in GBM cells

**Figure 6:**
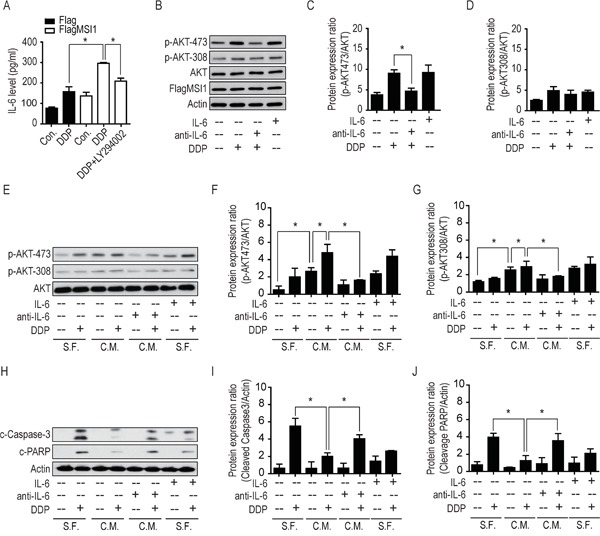
Musashi-1 mitigated DDP-induced apoptosis via the IL-6/AKT regulatory loop **A.** Cells were pretreated with 50 μM of LY294002 or vehicle for 3 hours, followed by 50 μM DDP treatment for 24 hours. The culture media were collected and the concentrations of IL-6 was determined by ELISA. **B.** 05MG-FlagMSI1 cells were treated with/without 50 μM DDP for 24 hours in the absence or presence of recombinant IL-6 (10 ng/ml) or anti-IL-6 neutralizing antibody (0.1 μg/ml). The cell lysates were analyzed by Western blot. **C-D.** The bar diagrams represent the quantified ratio of p-AKT-308/AKT and p-AKT-473/AKT obtained by the Western blot in B. **E.** 05MG cells were treated with control (serum free medium, S.F.) or condition media from 05MG-FlagMSI1 cells (C.M.) in the presence or absence of 50 μM DDP, IL-6 (10 ng/ml), and anti-IL-6 antibody (0.1 μg/ml) for 24 hours. The cell lysates were analyzed by Western blot to assess the levels of p-AKT-308, p-AKT-473, and total AKT. **F-G.** The bar diagrams represent the quantified ratio of p-AKT-308/AKT and p-AKT-473/AKT obtained in E. **H.** 05MG cells were treated as described in E and analyzed by Western blot. **I-J.** The bar diagrams represent the quantified ratio of cleavage-caspase-3/actin and cleaved-PARP/actin obtained in H. Data represent the mean ± S.D. of two independent experiments performed in triplicate. * P<0.05 vs AKT or Actin.

IL-6 exhibits a central role in host defense against infection and tissue injury, as well as in the progression of cancer malignancy [29]. IL-6 have been demonstrated to regulate the self-renewal in breast CSCs [30, 31], triggering malignant features in ductal breast carcinoma [31] and mediating drug resistance in breast cancer cells by expanding CSC population [32]. Robust pro-inflammatory studies have revealed that IL-6 intermediates the AKT/PI3K pathway in contribution to cancer cell survival [33–35], but the connection with MSI1 remain uncertain. Since we have demonstrated the increase of IL-6 biogenesis in MSI1-overexpressed cells with DDP treatment, secretory IL-6 level also elevated in consistence to mRNA expression using ELISA examination (Figure 6A). Concurrently, the exogenous IL-6 induced the phosphorylation of AKT on Ser473 rather than Thr308 (Figure 6B-6D). To further dissect the microenvironmental change, we treated the GBM cells with the conditioned medium (C.M.) comparing to serum-free medium (S.F.), suggesting the secretory fact of IL-6 in stressful MSI1-overexpressed GBM cells while anti- IL-6 could effectively neutralize the effect (Figure 6E-6G). According to our data, the condition medium from DDP-treated cells did activate AKT phosphorylation in the recipient cells; moreover, anti-IL-6 neutralizing agents impaired the DDP-induced AKT phosphorylation, indicating that IL-6 signaling cascade acts as a leading role in an autocrine/paracrine fashion. Coherently, the autocrinal/paracrinal IL-6 was found to cooperate with AKT in elevating anti-apoptotic events (Figure 6H-6J). Collectively, our data illustrated the autocrinal/paracrinal anti-apoptotic circuit that MSI1 activates AKT for IL-6 biogenesis and secretion, which in turn, autonomously activates AKT at Ser473 phosphorylation.

### Musashi-1 promotes *in vivo* tumor growth of GBM cells and IL-6 autocrine/paracrine secretion in tumor microenvironment

**Figure 7:**
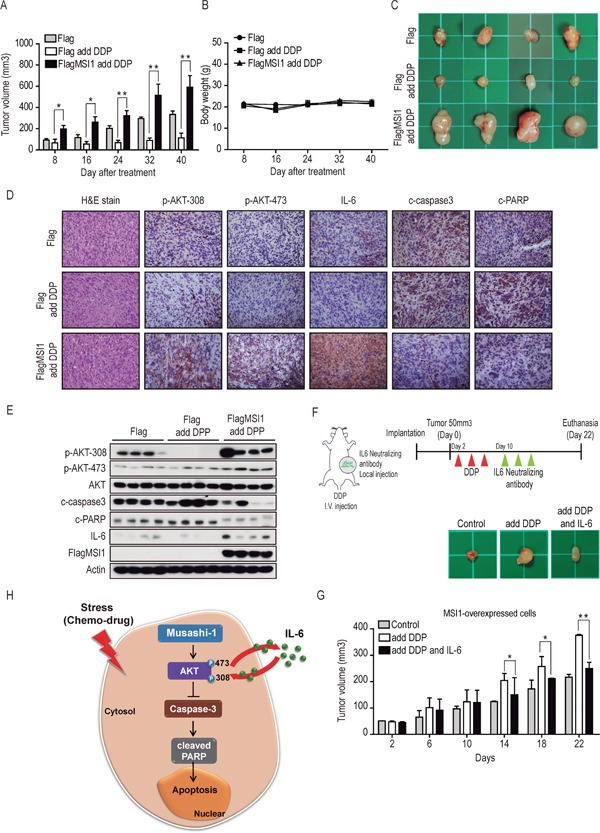
Musashi-1 promoted tumor growth and mitigates stress-induce apoptosis in xenograft animal model **A.** Nude mice were subcutaneously transplanted with 05MG-Flag or 05MG-FlagMSI1 cells and were administrated with DDP (20 mg/kg) or PBS through tail vein injection 3 weeks after transplantation, 3 times with 2-day intervals. The tumor size was measure with a caliper. Data were presented as mean±S.D. (n=12). Tumor size of mice with MSI1 overexpressed cells were significantly higher than those of Flag-control mice under DDP treatment (*P<0.05 or **P<0.01). **B.** Change of mice body weight during the 40 days of observation after DDP administration. **C.** Xenograft tumors were excised 40 days after DDP treatment. **D.** Tumor tissue were sectioned and subjected to H.E. staining and immunohistochemistry analysis to evaluate p-AKT-308, p-AKT-473, IL-6, cleaved-caspase-3 and cleaved-PARP expression levels. **E.** Tumors tissue (four of each group) were harvested and homogenized. Whole-tumor lysates were analyzed by Western blot analysis. **F.** Schematic illustration depicts the experimental design. Nude mice were subcutaneously transplanted with MSI1-overexpressed cells and were administered with DDP (20 mg/kg) or PBS intravenously 3 weeks after transplantation, 3 times with 2-day intervals. After DDP injection, tumors were administered with IL-6 neutralizing antibody (0.15μg/ml) with intratumoral injection, 3 times with 2-day intervals; **G.** The tumor sizes were measured with a caliper. Data were mean S.D. (n=3). Statistical analysis was carried out by Student's t-test (*P<0.05 or **P<0.01). **H.** A schematic diagram of the MSI1-dependent regulation of cell survival in GBM. In this model, MSI1 enhance AKT phosphorylation, leading to increased IL-6 secretion and decreased caspase3 and PARP activity, protecting GBM cell from chemodrug-induced apoptosis. The feedback regulation between AKT and IL6 augments the anti-apoptotic effect of MSI and maintains cells in a state of less sensitive to chemodrugs.

It has been shown in animal model that enhanced production of IL-6 may increase inflammation and tumorigenesis in cancer [36], and MSI promotes cell proliferation and cancer growth of colorectal carcinoma [7]. We have demonstrated the *in vitro* tumorigenicity as well as colony forming ability of MSI1-overexpressed GBM cells in Figure 1. The *in vivo* effects of MSI1 in chemoresistance and autocrinal/paracrinal status, however, still remain an open question. We therefore subcutaneously inoculated the Flag-control cells or MSI1-overexpressed GBM cells in nude mice and treated them with vehicle of DDP (10 mg/kg intraperitoneally) to determine if our findings were interpretable from *in vitro* to *in vivo* study. DDP modestly, but significantly promoted tumor growth at 8 days after the first drug exposure (Figure 7A and 7C; *P*<0.05). The effect become more apparent after 24 days (Figure 7A and 7C; *P*<0.01). No statistically significant changes in body weight were noted between the control and DPP-treated mice (Figure 7B). These data suggested that the overexpression of MSI1 overturned the killing effect of DDP and promoted the growth of tumor.

To further evaluate the levels of microenvironmental IL-6 and cellular active PARP and Caspase-3, we performed immunohistochemistry (IHC) staining of tumor tissues. Consistent to our *in vitro* results, overexpression of MSI1 in GBM enormously increase the infiltration of pro-inflammatory factor IL-6 throughout the tissue section (Figure 7D). Besides, the pro-apoptotic proteins, including caspase3 and PARP, were decreased in the DDP-treated 05MG-FlagMSI1 xenograft tumors, indicated the chemoresistance in MSI1-overexpressed GBM tissue (Figure 7D). In addition to IHC, we extracted the proteins from four individual mice in each group, demonstrating the synchronous pattern between IL-6 level and AKT phosphorylation (Figure 7E). Taken together, we collectively address the possible regulatory circuit that AKT activation takes reciprocal action to IL-6 biogenesis and secretion, while MSI1 plays a dominant role in initiating this anti-apoptotic process (Figure 7F). To address the autocrinal/paracrinal fashion in IL-6-mediated MSI1-tumorigenesis *in vivo*, we subcutaneously inoculated the MSI1-overexpressed cells in nude mice and treated them in the absence or presence of IL-6 neutralizing antibody (0.15μg/ml, i.p.). As shown in Figure 7G, DDP promoted tumor growth at the 10th day after the first DDP administration, but was then retarded by IL-6 neutralizing antibody treatment at the 14th day. Our data also suggested that overexpressed MSI1 induced IL-6 secretion and promoted tumor growth, nevertheless, ablation of IL-6 by the neutralizing antibody withdrawn the malignant phenotypes. These findings confirmed the MSI1-dependent suppression of apoptosis and induction of chemoresistance, as well as the MSI1-induced AKT activation and IL-6 autocrine/paracrine secretion in GBM tumor *in vivo*.

## DISCUSSION

Drug resistance has been declared the major concern along the anti-cancer treatment in a curative intent. The concept of anti-cancer drug is preferentially to enforce the cancer cells into programmed cell death, but the anti-apoptotic signal has forfeited the process and eventually results in multiple drug resistance. Regarding MSI1 was found highly expressed in human central nervous system and considered as an essential marker for neural stem cells or progenitor cells, accumulated studies have indicated MSI1 enriches the population of cancer stem cells [2, 37, 38]. For instance, Sureban et al. reported the correlation between MSI1 and radio-resistance in human colon cancer cells [7]. Besides, MSI1 is known for functioning as translation regulators from targeting mRNAs and is critical in maintenance of stemness and self-renewal capability [39, 40]; meanwhile, the correlation between highly expressed MSI1 and tumor malignancies has been verified in breast cancer, endometrial carcinoma and adenocarcinoma [41–43]. Although MSI1 expression has been hinted as a prognosis factor in various cancers [44–46], the underlying mechanism involved in drug resistance is still an open question. Here, we address that MSI1 protect tumor cells from drug-induced apoptosis and promote tumor growth. Blockade of AKT phosphorylation by LY294002, a PI3K inhibitor, greatly relieved the activation of caspase-3 and PARP signaling in MSI1-overexpressed GBM cells. In fact, MSI1 secures the cells from apoptotic process, and in part, increases the growth of cancer cells and clonogenic ability. Global gene expression array indicated that MSI1 post-translationally activates AKT through PI3K-dependent phosphorylation, which triggers the survival signaling in the downstream and vice versa.

AKT signaling pathway has long been a well-studied cell-fate pathway regulating the apoptosis and survival genes, including ERK, BAD, Forkhead, etc. However, little is known about the regulation between MSI1 and AKT, despite Muto et al. reported that MSI1 knockdown reduced the activity of Akt-PI3K and NOTCH pathway in response to the directly posttranscriptional regulation of Numb and PTEN [8]. AKT contains two phosphorylation sites that are in favor of different kinases, where serine 473 (Ser473) and threonine 308 (Thr308) are targeted by mTORC2 and 3-phosphoinositide dependent kinase 1 (PDK1), respectively [20]. Although some reports indicated that Thr308 retains more reliability than Ser473 of being a prognostic factor in non-small cell lung cancer and leukemia [47, 48], Ser473 is likely to perform more contribution in cellular survival regulation [49, 50]. Therefore, we coordinately examined the two phosphorylation sties in response to chemodrugs with MSI1 modulation. We demonstrate that MSI1 dominantly activates AKT through Ser473 phosphorylation than that of Thr308, suggesting direct correlation between MSI1 and Ser473 controls drug-induced apoptosis, which acts as a protective mechanism in stressful cells. However, how the phosphorylation of AKT is regulated by MSI1 is still remain mystery. In order to dissect the putative regulatory path, we conducted a systemic overview on our microarray results, and identified that TNS2 mRNA was decreased in MSI1 overexpressed cells (data not show). TNS2 inhibits AKT/PKB signaling via C1-TEN (C1 domain-containing phosphatase and TENsin homologue) and mediates TPO (Thrombopoietin)/c-Mpl pathway [51], which engages AKT signaling in induction of cancer growth [52]. Thus, we imply that MSI1 is responsible for modulating AKT phosphorylation through multiple indirect ways, including posttranscriptional down-regulation of Numb/PTEN and TNS2, and eventually achieve the survival of cancer cells under chemotherapy. Moreover, our microarray data additionally suggested that the pro-inflammatory related gene clusters were highly enriched, which may as well attribute the microenvironment for GBM cells to not only maintain the self-renewal stemness but also the differentiating plasticity. For instance, Leibovich-Rivkin et al. displayed that the pro-inflammatory microenvironment despite induce the malignant transformation of normal breast epithelial cells, those secreted cytokines also provide an excellent scaffold for circulating cancer cells to be seeded and migrated [53].

Interleukin-6 (IL-6), a pro-inflammatory cytokines in host defense against inflammation and tissue injury, has been linked to tumorigenesis, angiogenesis, and survival in malignant tumors like GBM [29]. The role of IL-6 as critical mediator for tumor growth, metastasis, and drug resistance in tumor micro-environment was therefore suggested [54]. For instance, IL-6 gene amplification has been linked to GBM aggressiveness and poor patient survival [55, 56]. Both of transgenic animals and *in vitro* investigations reported that overexpression of the canonical IL-6 signaling pathway is a major characteristic in human neoplasia and that oncogenic overexpression of this pathway can occur at many levels [57, 58]. Furthermore, IL6-mediated suppression of miR200c drives the transformation of breast cancer cells [59], and activation of an IL6 inflammatory feedback loop leads to expansion of cancer stem cell population in malignant carcinomas [32]. In addition, IL-6 was shown to induce breast cancer stem cell formation from non-stem cancer cells through an autocrine/paracrine mechanism [30] and to regulate epigenetic modification of microRNAs in malignant human cholangiocarcinoma [60]. It is noteworthy that Chiou et al. reported a paracrine feedback regulatory loop between miR142-3p and IL-6 in GBM cells [33], in which IL-6 suppresses miR142-3p expression through increasing its promoter methylation, while miR142-3p inhibits IL-6 expression and secretion by directly targeting IL-6 3′UTR. Additionally, AKT is previously reported to play positive role in regulating IL-6 biogenesis and secretion by NF-kappaB and MAPK pathway, supporting that AKT may upstream modulator in IL-6 pro-inflammatory pathway [61, 62]. Although the definition of autocrine and paracrine differs from each other, our solid evidence demonstrates the paracrinal effect of DDP-treated GBM cells but the autocrinal effect is unable to be ruled out from our results. Regarding the previous studies, the IL-6 regulatory circuit can be jointly categorized as autocrine/paracrine rather than an individual one [63–67]. Additionally, AKT is previously reported to play positive role in regulating IL-6 biogenesis and secretion by NF-kB and MAPK pathway, supporting the notion that AKT may upstream modulator in IL-6 pro-inflammatory pathway [61, 62]. Recently, IL-6 is reported to play a positive role in regulating AKT/PI3K activity to promote tumor cell survival [68]. Direct correlation between IL6, AKT and NF-kB reduced increased tumor growth and metastasis in mouse xenografts since IL-6 feedback loop, which is also validated as a protective mechanism in cells under stress condition [32]. Our *in vitro* results indicated a regulatory circuit between AKT and IL-6, in which autocrinal/paracrinal IL-6 increased AKT activity while phosphorylated AKT enhanced IL-6 secretion to the microenvironment. This circuit resulted in suppressed apoptotic molecules such as cleaved PARP and Caspase-3. Similarly, our *in vivo* results also indicated the infiltrated expression of IL-6 throughout the sectioned tumor tissue, emphasizing the potential autocrinal/paracrinal regulation. Therefore, the results inspired us to retreat the drug resistance through anti-IL-6 therapy on the basis of both epigenetic modulation and our displayed results of MSI1-induced AKT-IL-6 autonomous regulatory circuit.

Taken together, our study indicates the autonomous MSI1-dependent regulation of AKT and IL-6 in GBM, and partly answered the unsolved question of how MSI1 takes effect on autocrine/paracrine secretion, as well as tumor environmental pro-inflammatory biogenesis. Further studies may be required to investigate the aberrant mechanism of AKT and IL-6 in contribution to other chemotherapeutic agents as well as radiotherapy. These findings could be of valuable in applying anti-IL-6 to withdraw the drug resistance of cancer cells clinically.

## MATERIALS AND METHODS

### Cell culture

The human GBM cell line, 05MG, and its derived MSI1-overexpressed stable cell line were cultured in Dulbecco's Modified Eagle's Media (DMEM, Life Technologies Inc., Carlsbad, CA, USA) with 10% fetal bovine serum (HyClone Laboratories Inc., South Logan, UT, USA), 150 g/mL G418 (Life Technologies Inc., Carlsbad, CA, USA), 100 units/mL penicillin, and 100 μg/mL streptomycin (Life Technologies Inc., Carlsbad, CA, USA) under standard culture conditions (37°C, 95% humidified air and 5% CO2). Subcultures were performed with 0.25% trypsin-EDTA (Sigma-Aldrich Co. LLC., St. Louis, MI, USA). Media were refreshed every two days.

### Cell viability assay

Human GBM 05MG cells were seeded in 24-well plates (3000 cells per well) with complete growth medium. The medium was replaced by either solvent or chemicals with indicated concentrations in complete medium. Cell viability assay was then performed. In brief, cells were stained with 0.1 mg/ml 3- (4,5-cimethylthiazol-2-yl)-2,5-diphenyl tetrazolium bromide (MTT, Sigma-Aldrich co. LLC., St. Louis, MI, USA) for 2 hours and the formazon crytals were then dissolved in DMSO. The relative absorbance was then measured by TECAN Sunrise ELISA plate reader (Thermo Scientific Inc., Waltham, MA, USA) at 570 nm.

### Colony formation assay

GBM cells were seeded in 6-well plates (1,000 cells per well) and were incubated for 24 hours. The cells were subjected to chemodrug exposure for 24 hours (cisplatin, DDP) or 16 hours (arsenic trioxide, ATO) and the drugs were removed with growth media. Further 10-day incubation was performed, and the cells were fixed by 9% formalin, and stained by 4% trypan blue (w/v) for 20 min. The stained cells were washed using PBS and counted.

### Determination of apoptosis

Apoptotic events were determined by Annexin V (BD Pharmingen™, #556547), cleaved caspase-3 (BD Pharmingen™, #560901) and terminal deoxynucleotide transferase-mediated dUTP nick-end labeling (TUNEL) staining. For flow cytometry, cells were harvested and stained with both Annexin V and PI for 10 min. They were then washed by PBS, and resuspended in HEPES. For cleaved caspase-3 staining, deparaffinized sections were subjected to antigen retrieval in 0.01 mol/ml citrate buffer (pH 6.0) by microwave heating. After blocking with 5% Bovine Serum Albumin (BSA, Life Technologies Inc., Carlsbad, CA, USA), the sections were incubated with rabbit monoclonal anti–cleaved caspase-3 antibody (Cell Signaling Technologies Inc., Danvers, MA, USA). TUNEL assay was performed following the manufacturer's instructions. The nuclei were counterstained using DAPI. The number of positively stained nuclei was counted from 10 fields of view and 5 different groups.

### Western blotting

Protein samples were prepared with RIPA buffer (Thermo Scientific Inc., Waltham, MA, USA) containing 1% protease inhibitor. Equal weight of total protein was separated by electrophoresis on SDS/PAGE. After the proteins had been transferred onto a polyvinylidene difluoride membrane (Millipore, Bedford, MA, USA), the blots were incubated with blocking buffer (1 X PBST and 5% skim milk) for 1 hour at room temperature and then hybridized with primary antibodies overnight at 4°C, followed by incubation with horseradish peroxidase-conjugated secondary antibody for 1 hour at room temperature. The blots were obtained by X-ray film exposure, and the intensities were quantified by densitometry analysis (Digital Protein DNA Imagineware, Huntington Station, NY).

The primary antibodies used in this study were anti-Flag (Sigma-Aldrich Co. LLC., St. Louis, MI, USA, F1804), AKT (cell signaling, #9272), p-Akt-308 (cell signaling, #13038), p-AKT-473 (cell signaling, #4060), Bad (cell signaling, #9239), p-Bad (cell signaling, #5284), Bax (cell signaling, #2772), cleaved-caspase-3 (cell signaling, #9661), cleaved-PARP (cell signaling, #5625), IL-6 (R&D system, #6708) and beta-actin (sigma, A5316).

### Quantitative real-time PCR (qRT-PCR)

Total RNA were isolated from GBM cells using TRlzol (Life Technologies Inc., Carlsbad, CA, USA) followed by phenol:chloroform purification and ethanol precipitation. Reverse transcription were carried out using SuperScript III reverse transcriptase (Life Technologies Inc., Carlsbad, CA, USA). Oligonucleotides (Suppl. Table 1) were designed using Primer Express 2.0 (Applied Biosystems, Foster City, CA, USA). Oligonucleotide specificity was computer tested (BLAST, National Center for Biotechnology Information, Bethesda, MD, USA) by homology search with the human genome and later confirmed by dissociation curve analysis. The qRT-PCR was performed with power SYBR Green PCR Master Mix (Applied Biosystems, Foster City, CA, USA) according to manufacturer's instruction. Signals were detected with 7900HT Fast Real-time PCR system (Applied Biosystems, Foster City, CA, USA). The expression level of each gene was normalized to endogenous beta-actin and experimental control through ΔCt methods.

### Gene expression array and bioinformatic analysis

Total RNA was extracted as previous description and the extracts were subjected to hybridized with Agilent SurePrint G3 human whole genome gene expression chip (Agilent Technologies, Santa Clara, CA, USA). Gene expression array data Genetic network construction was performed by Ingenuity Pathway Analysis (IPA) software (Qiagen, Hilden, Germany). The cutoff was performed by 1.5 log2 ratio and 989 genes were annotated as gene list for further analysis. Genetic function and gene enrichment analysis were concluded by DAVID Bioinformatics Resources (http://david.abcc.ncifcrf.gov/). The gene ontology (GO) terms was further summarized by REVIGO (http://revigo.irb.hr/).

### siRNA transfection

MSI1 and scrambled control were purchased from GE Dharmacon On-TARGETplus siRNA smart pools. Transient transfection was carried out using INTERFERin siRNA transfection reagent (Polyplus Transfection, Huntingdon, UK) according to the manufacturer's instruction. Cell-based experiments were performed after 2-day incubation.

### Plasmid DNA transfection

Plasmid transfection was carried out using jetPEI DNA transfection reagent (Polyplus Transfection, Huntingdon, UK) according to manufacturer's instructions.

### Cytokine ELISA

The GBM cells were stimulated with cisplatin for 24 hours with or without LY294002 (PI3K inhibitor, Merck KGaA, Darmstadt, Germany). Human IL-6 (R & D system, # D6050) from either the supernatants or the serum were measured by ELISA according to the manufacturer's instructions.

### Animals and tumor cell transplantation

All procedures involving animals were performed in accordance with the institutional animal welfare guidelines of Taipei Veterans General Hospital. The GBM cell line 05MG-Flag and 05MG-MSI1 were harvested, washed, resuspended in PBS and subjected to be subcutaneously implanted into the dorsolateral side of the flank region of 8-week-old male BALB/c nude mice(National laboratory animal center, Taipei Taiwan). After 14 days of subcutaneous inoculation. The treating regimen of cisplatin (2 mg/kg) was designated by q3dx4 (every 3 days for four i.v. injection) to mimic clinical chemotherapy. Tumor volume was measured by calipers. Tumor size in the subcutaneous xenograft model was measured every two days using a caliper. The average tumor volume was calculated using the following equation: V= A x B^2^ x 0.5 (A, long diameter; B, short diameter).

### Immunohistochemistry staining and immunoblotting (IHC)

Tumor specimens from mice were fixed with 4% paraformaldehyde (Sigma-Aldrich Co., St. Louis, MI, USA). Sections were deparaffinized and rehydrated, and subjected to antigen retrieval by boiling in 10 mmol/L (pH 6) citrate buffer (Sigma-Aldrich Co., St. Louis, MI, USA) for 10 mins. Sections were cooled in PBS for 10 mins before treating with 3% H_2_O_2._ Sample were blocked in 5 mg/ml BSA (Sigma-Aldrich Co., St. Louis, MI, USA) for 30 mins before hybridizing with 1/100 diluted primary antibodies p-Akt-308 (cell signaling, #13038), p-AKT-473 (cell signaling, #4060), cleaved-caspase-3 (cell signaling, #9661), cleaved-PARP (cell signaling, #5625) and IL-6 (R&D system, #6708) overnight at 4°C. Signals were amplified by the TSA Biotin System (PerkinElmer, Waltham, MA) following the manufacturer's instruction and the samples were counterstained with hematoxylin. The sections were examined under Olympus BX61 microscope (Olympus Corp., Tokyo, Japan), and three field of views were randomly selected and photographed for evaluation. The relative staining index (rSI) represents the percentage of positive-expressed cells in the counting region.

### Data analysis

Data are expressed as the mean ± SD from at least three independent experiments. The statistical analysis was performed using student's T-test. Difference were considered significant when p ≤ 0.05 or p ≤ 0.01.

## SUPPLEMENTARY FIGURES AND TABLE


