# Supplementary material for: Musashi-1 regulates AKT-derived IL-6 autocrinal/paracrinal malignancy and chemoresistance in glioblastoma
Source: Oncotarget. 2016 Jun 7;7(27):42485–501. doi: 10.18632/oncotarget.9890 (PMC5173150; doi:10.18632/oncotarget.9890)
Supplement: Supplementary file 1 [file oncotarget-07-42485-s001.pdf]

Musashi-1 regulates AKT-derived IL-6 autocrinal/paracrinal malignancy and chemoresistance in glioblastoma

SUPPLEMENTARY FIGURES AND TABLE

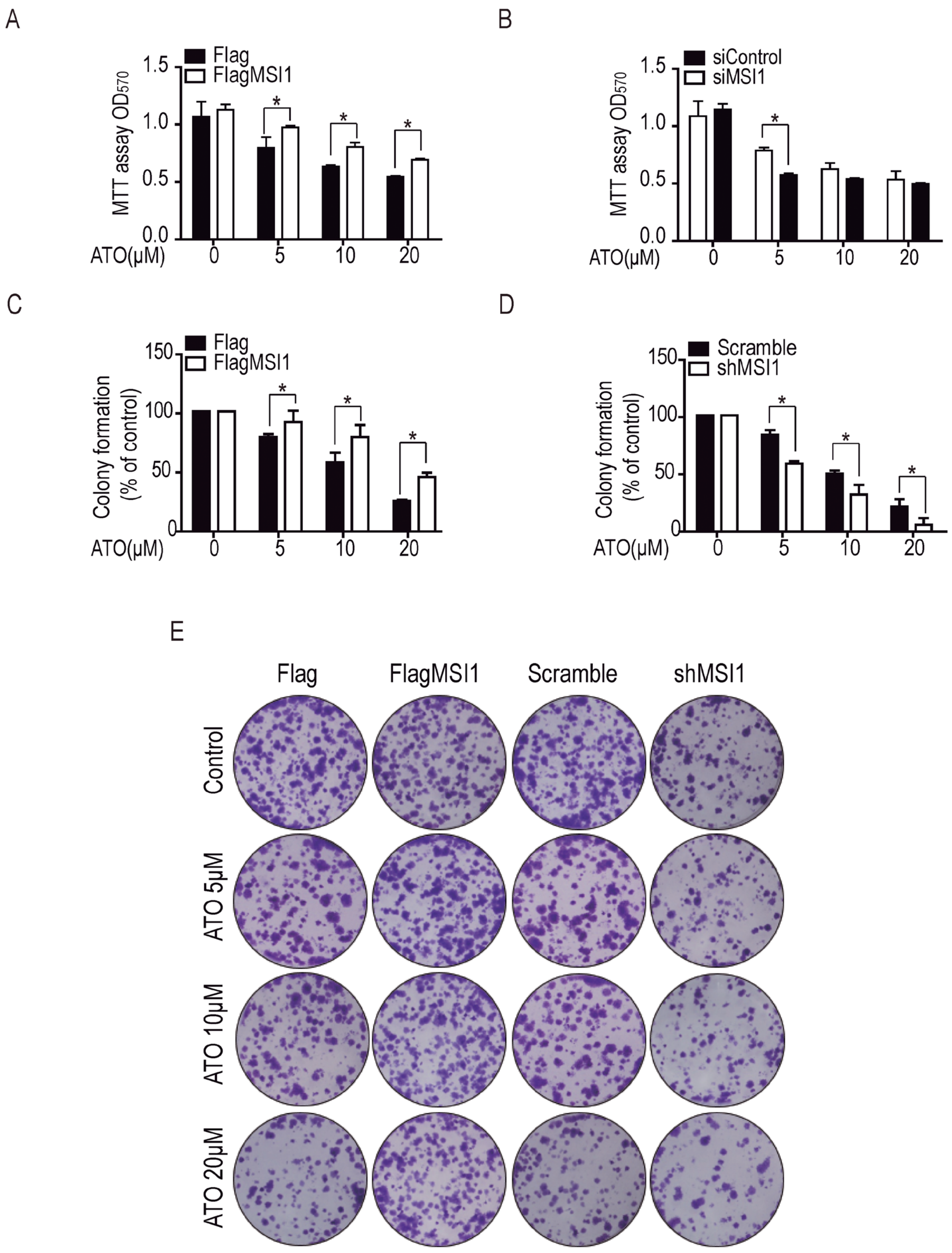

Supplementary Figure S1: Musashi-1 enhanced cell viability and colony formation under ATO treatment in GBM cells. A-B. Cells were treated with a dose-course ATO (from 0 to 20 μM) for 16 hours, and the cell viability was determined by MTT assay. **C-D.** Cells were treated with a dose-course ATO for 16 hours, followed by a colony formation assay incubated for 10 days allowing the survived cells forming colonies. Data represent the mean ± S.D. of three independent experiments performed in triplicate. * $P<0.05$ vs control cells. **E.** The formation of colonies in C and D was photographed.

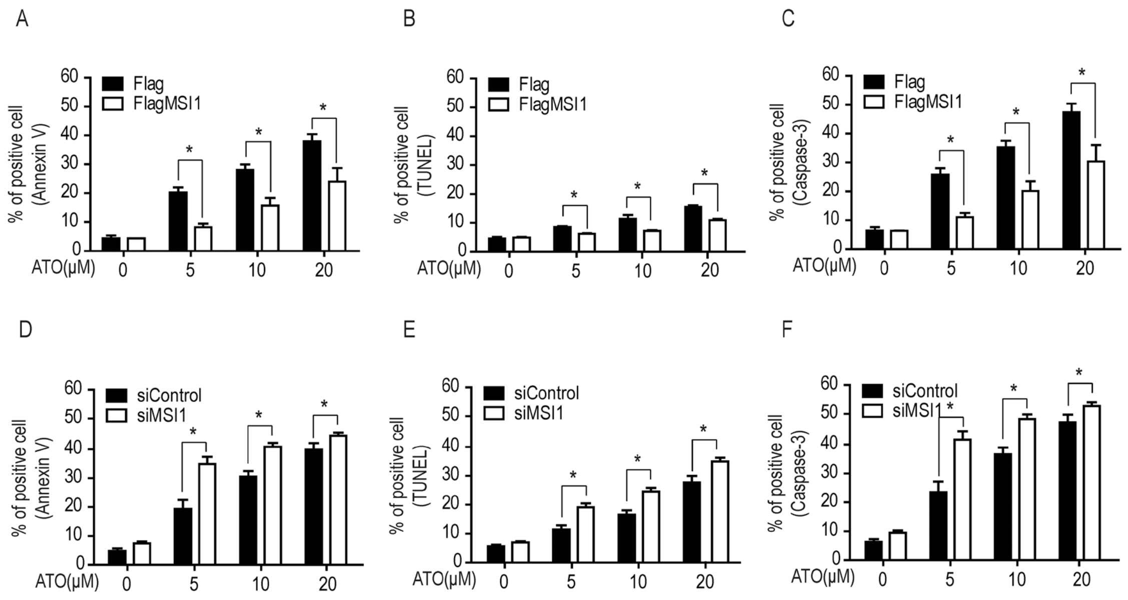

Supplementary Figure S2: Musashi-1 mitigated ATO-induced apoptosis in GBM cells. MSI-overexpressed (A-C) and knockdown (B-F) cells were treated by a dose-course of ATO for 16 hours. The apoptotic cells were identified by the staining of AnnxinV (A, D), TUNEL assay (B, E), and activated caspase-3 (C, F), and quantified by flow cytometer. Data represent the mean ± S.D. of three independent experiments performed in triplicate. * P<0.05 vs control cells.

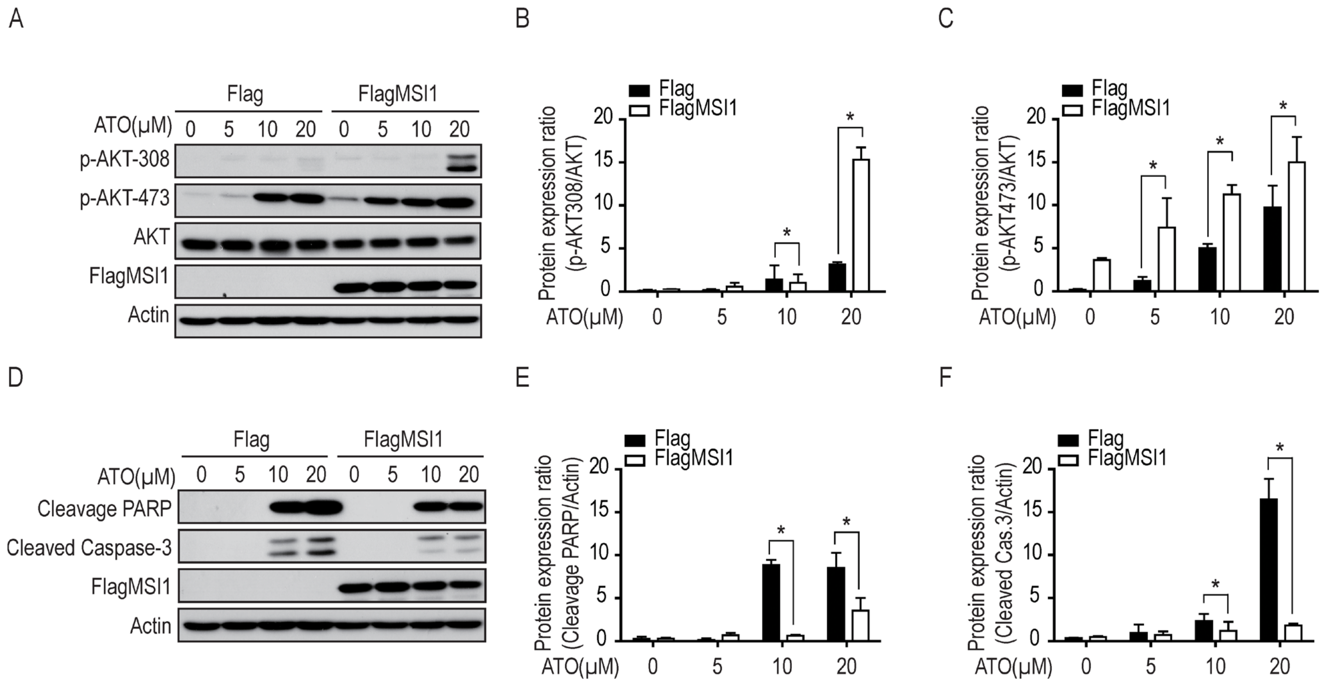

Supplementary Figure S3: Musashi-1 enhanced AKT phosphorylation and reduced the levels of cleaved-caspase-3 and cleavage-PARP under DDP treatment. A-C. 05MG-FlagMSI1 and 05MG-Flag cells were treated with different concentration of ATO for 16 hours. Total lysates were analyzed by Western blot to assess the level of phosphorylated and total AKT. **B.** Relative ratio of p-AKT308/AKT was quantified and presented in the bar chart. **C.** Relative ratio of p-AKT473/AKT. **D.** The same experiment as described in A was performed to assess the expression level of cleaved PARP and Caspase-3. **E.** Relative ratio of cleaved-PARP/actin. **F.** Relative ratio of cleaved-Caspase-3/actin. Data represent the mean ± S.D. of three independent experiments performed in triplicate. * $P<0.05$.

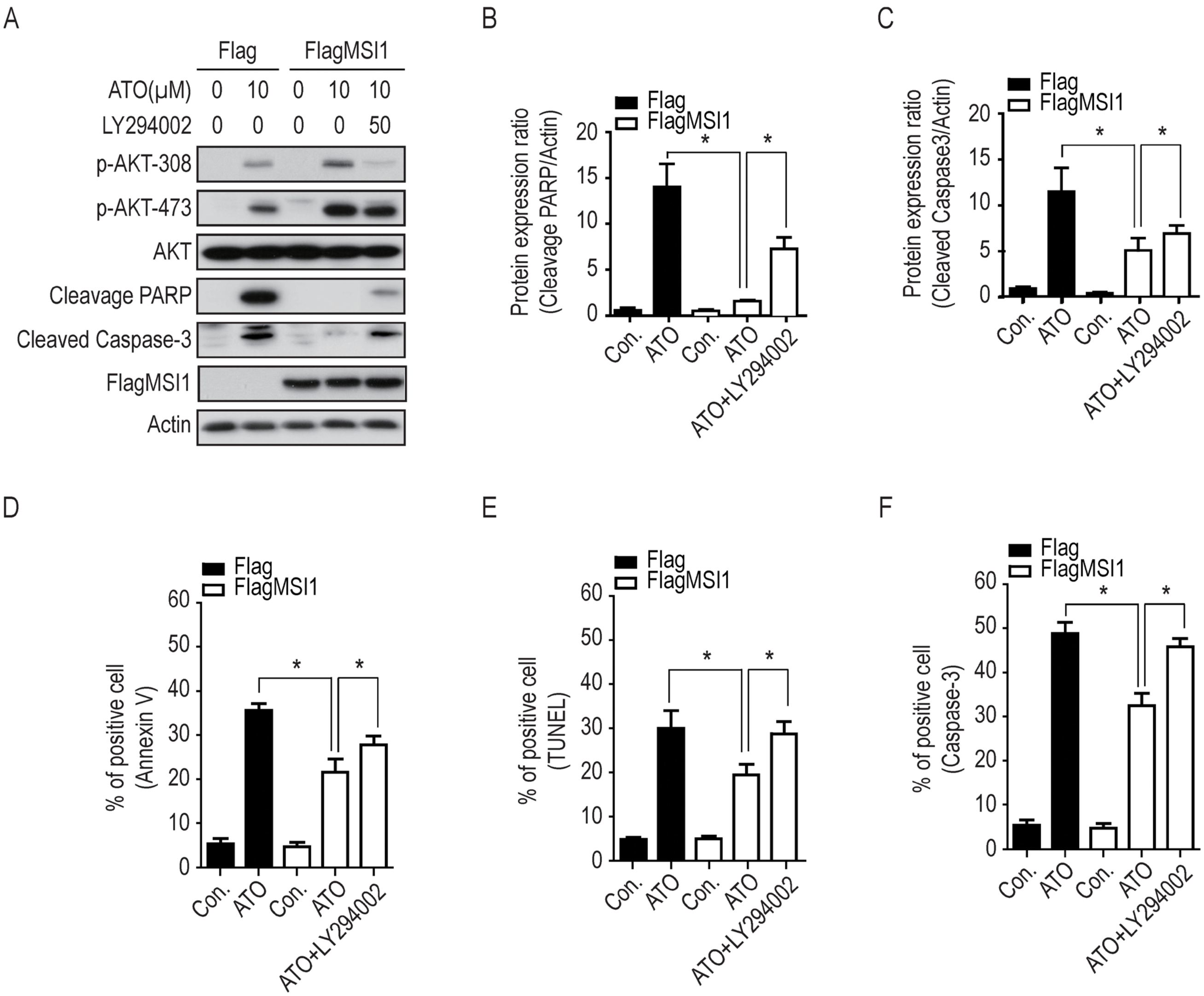

Supplementary Figure S4: Inhibition of AKT activity blocked the suppressive effect of MSI1 on ATO-induced apoptosis. 05MG-FlagMSI1 and 05MG-Flag cells were pretreated with 50 µM of LY294002 or vehicle for 3 hours, followed by 10 µM ATO treatment for additional 16 hours. **A.** Total cellular extracts were analyzed by Western blot analysis. **B.** Quantified cleaved PARP protein levels in A were standardized with actin level. **C.** Quantified cleaved Caspase-3 protein levels in A were standardized with actin level. Data represent the mean ± S.D. of three independent experiments performed in triplicate. * P<0.05. **D-F.** Percentage of apoptotic cells were quantified by Flow cytometry in Annexin V (D), TUNEL (E), and Caspase-3 (F) assay. Data represent the mean ± S.D. of three independent experiments performed in triplicate.

Supplementary Table S1: List of primers used in quantitative real-time PCR

Gene Name	Forward	Reverse
IL-6	GAACTCCTTCTCCACAAGCG	CTGAAGAGGTGAGTGGCTGTC
IL-7	AACTGCACTGGCCAGGTTAAA	GGATGCAGCTAAAGTTCGTGT
IL-17	CCTTGGAATCTCCACCGCAA	GACAATCGGGGTGACACAGG
BICR3	CCAAGTGGTTTCCAAGGTGTG	TAAAGCCCATTTCCACGGCA
CXCR4	GGTTACCATGGAGGGGATCAG	GGTGCAGCCTGTACTTGTCC
CXCL3	TGTGAATGTAAGGTCCCCCG	ACCCTGCAGGAAGTGTCAAT
